# Changes of endophytic microbial community in *Rhododendron simsii* roots under heat stress and its correlation with leaf physiological indicators

**DOI:** 10.3389/fmicb.2022.1006686

**Published:** 2022-11-17

**Authors:** Wei Lin, Lei Liu, Jincheng Liang, Xuexiao Tang, Jie Shi, Li Zhang, Purui Wu, Siren Lan, Shusheng Wang, Yan Zhou, XiaoChou Chen, Ying Zhao, Xiang Chen, Binghua Wu, Lijin Guo

**Affiliations:** ^1^College of Forestry, Fujian Agriculture and Forestry University, Fuzhou, China; ^2^Key Laboratory of Genetics and Germplasm Innovation of Tropical Special Forest Trees and Ornamental Plants, Ministry of Education/College of Forestry, Hainan University, Haikou, China; ^3^State Key Laboratory of Hydraulic Engineering Simulation and Safety, School of Civil Engineering, Tianjin University, Tianjin, China; ^4^College of Tropical Crops, Hainan University, Haikou, China; ^5^Lushan Botanical Garden, Jiangxi Province and Chinese Academy of Sciences, Lushan, China; ^6^Guizhou Botanical Garden, Guiyang, China; ^7^Fuzhou Botanical Garden, Fuzhou, China; ^8^Institute of Biology, Guizhou Academy of Sciences, Guiyang, China; ^9^College of Horticulture, Fujian Agriculture and Forestry University, Fuzhou, China; ^10^International Magnesium Institute, College of Resources and Environment, Fujian Agriculture and Forestry University, Fuzhou, China

**Keywords:** *Rhododendron simsii*, heat stress, endophytic microorganism, physiological characteristics, sequencing

## Abstract

**Introduction:**

The response mechanism of Rhododendron simsii and its endophytic microorganism to heat stress is still unclear.

**Methods:**

The light incubator was used to set the temperature gradients, and the control (CK) was (day/night: 14/10 h) 25/22°C, the moderate-heat-stress (MHS) was 35/30°C and the high-heat-stress (HHS) was 40/35°C.

**Results:**

Compared with CK, MHS significantly increased the contents of malondialdehyde, hydrogen peroxide, proline, and soluble sugar, as well as the activities of catalase and peroxidase in leaf, while HHS increased the activities of ascorbate peroxidase, and decreased chlorophyll content. Compared with CK, MHS reduced soil available nitrogen (N) content. Both heat stress changed the endophytic microbial community structure in roots. MHS enriched Pezicula and Paracoccus, while HHS significantly enriched Acidothermus and Haliangium. The abundance of Pezicula positively correlated with the contents of chlorophyll a and proline in leaf, and negatively correlated with soil ammonium N content. The abundance of Pezicula and Haliangium positively correlated with soluble sugar and malondialdehyde contents, respectively.

**Conclusions:**

Our results suggest that root endophytic microorganisms play an important role in helping Rhododendron resisting heat stress, mainly by regulating soil N content and plant physiological characteristics.

## Introduction

*Rhododendron* spp. is a large group of Ericaceae, which is well-known for its highly-presentable flowers commonly used for horticultural display (Li et al., 2021; Liu, L. et al., 2022). In recent years, its value on medicinal uses been studied, and its character of ecological friendly have also been recognized (Lin et al., 2019; Li et al., 2021). Although China owns more than half of the world’s species of *Rhododendron* spp. (Yu et al., 2017), the plant cannot be utilized on urban garden greening on a large scale across the country for several special reasons. First, heat stress can affect plant growth and development considered a major factor that limits the application of *Rhododendron* spp. in urban gardening (Chaudhry and Sidhu, 2021). Second, heat stress can affect the fluidity of cell membrane in the plant, and even damage cell membrane directly (Luo et al., 2021). Electrolyte leak and lipid composition changes, which caused by the destruction of cell membrane, could lead to the accumulation of reactive oxygen species in plants, thus reducing osmotic potential and water conduction of cells, and eventually let it out of control (Jahan et al., 2019). Recently, researches focus on plants synthesizing antioxidants, such as superoxide dismutase, peroxidase (POD), catalase (CAT) and ascorbate peroxidase (APX), functional substances that remove excess reactive oxygen species from cells (Farooq et al., 2021).

In addition, plants also produce osmotic regulatory substances such as proline (Pro), soluble sugar, and soluble protein to regulate the water relationship inside the plants in order to resist heat stress (Hosseinifard et al., 2022). Under mild heat stress, the plants might self-regulate through a series of reactions, but high-heat stress often leads to protein denaturation inside the plants to self-regulation. Nevertheless, during the photosynthetic process of the plants, the main destructed site of the photosynthetic mechanism is photosystem II, high temperature leads to the inactivation of photosynthetic proteins, which has a negative impact on the synthesis of photosynthetic pigments, and makes plants prone to appear yellow leaves under heat stress (Xi et al., 2020; Liu, L. et al., 2022).

Nitrogen (N), is one of the components of proteins inside the plants, where the protein cannot be formed without N (Wen et al., 2020). In addition, plants take up N to synthesize chlorophyll and other organic matter necessary for plants. Therefore, N is naturally essential to the plants (Callbeck et al., 2021). Plants absorb inorganic N into their bodies through their roots and use that to maintain the normal plant growth and metabolism process through photosynthesis, including chlorophyll and protein (Wen et al., 2020; Qiu et al., 2021). Heat stress can affect the above-ground parts of the plant as well as the below-ground parts. Studies have shown that heat stress significantly reduced the content of carbon, N, and phosphorus in plant rhizosphere soil, resulted in a decrease of plant biomass (Khan et al., 2021). We also found that heat stress can change soil available N content, to influence the absorption of nutrients by plants, brought changes to various plant physio-logical indicators (Guo et al., 2022; Liu, L. et al., 2022).

Endophytic microbes, which includes bacteria and fungi, inhabit in plant’s root, which can benefit plant growth directly or indirectly (Vurukonda et al., 2018). While plants provide carbon sources for endophytic microorganisms to grow their own cellulose, endophytes improve the resistance to diseases and reduce abiotic stress of host plants (Khare et al., 2018). The invasion of living plant tissues by beneficial root endophytic microorganisms usually causes nonpathogenic infections and has good effects on plant grown (Dickie et al., 2010). Most endophytic bacteria are facultative bacteria, which can survive in both roots and soil (Woźniak et al., 2019). Therefore, in most cases, the species of endophytic bacteria in roots are similar to those in soil bacteria. *Pseudomonas*, *Enterobacter*, and *Agrobacterium* are common genera (Fouda et al., 2021). Most endophytic fungi that form symbiotic relationships with plants can only live in plants and are difficult to survive in soil alone (Rajamani et al., 2018). *Rhododendron* spp. usually grow in acidic and poor soil, along with thin, hairless roots. It relies mostly on endophytic fungi to help them absorb nutrients (Perotto et al., 2018). Therefore, the growth and development of *Rhododendron* spp. growth seem very dependent on endophytic microorganisms in root. Although the effects of these microorganisms on the structure and spatial distribution of root microbial communities of *Rhododendron* spp. and other plants have been studied (Fadaei et al., 2020; Calevo et al., 2021). However, most studies focus on single or mixed mycorrhizal fungi in the plants which effect to heat tolerance (Fadaei et al., 2020; Calevo et al., 2021). Yet, no studies have investigated the effects of heat stress on endophytic bacterial and fungal communities of *Rhododendron* spp. Bisht et al. (2020) showed that *B. Cereus SA1* significantly increased the pigment content and antioxidant enzyme activity in soybean, which alleviated the heat stress of soybean. Park et al. (2017) showed that endophytic microorganism *SA187* could significantly improve the biomass and grain yield of Arabidopsis thaliana and wheat under high temperature stress in a long-term field experiment. In recent years, there have been more and more studies on microbial mitigation of plant heat stress, but more attention is needed. In this study, *R. simsii* is selected as the subject throughout the research to solve the following two scientific questions: (1) what is the effect of heat stress on root endophytic microbial community? (2) what is the relationship between root endophytic microorganism, plant physiological characteristics, and soil physical–chemical properties under heat stress?

## Materials and methods

### Plant materials

The cutting seedlings of one-year-old *R. simsii* with strong growth, no disease, and insect pests, and consistent growth were selected as experimental materials. The test material was provided by Fujian Zhangzhou Jianhui Seedling Co., Ltd. Cutting seedlings of *R. simsii* were grown in nursery. Before temperature treatment, the cuttings or seeding had an average height of 20 cm and an average stem diameter of 0.49 cm. Klasmann peat (Geeste, Germany) was mixed with Perlite in a ratio of 3:1 and used as a cultivated soil. The physical and chemical properties of Klasmann peat are as follows: the pH is 4.25, water dissolved organic carbon content is 7.32 g kg^−1^, soil NH_4_^+^-N content is 285 mg kg^−1^, soil NO_3_^−^-N content is 8.82 mg kg^−1^, and soil microbial biomass N content is 24.12 mg kg^−1^. After the seedlings were transported to the artificial climate chamber, they were cultured under the same conditions (light intensity of 2,000 lx, day/night: 14/10 h, and relative humidity of 70–80%) for 1 month.

### Experimental design

The experiment was carried out for 3 months, which began in June 2021 and finished in August in the Key Laboratory of Genetics and Germplasm Innovation of Tropical Special Forest Trees and Ornamental Plants (Hainan University), Ministry of Education, Haikou city, Hainan province, China. Artificial climate chambers (CMP 6010,CONVIRON, Canada) were used to set the temperature gradients, and the control (CK) was (day/night: 14/10 h) 25/22°C, the moderate-heat-stress (MHS) was 35/30°C and the high-heat-stress (HHS) was 40/35°C. A completely randomized trial with four replications was used in this study. During the experiment, all the environmental conditions and management measures except temperature were kept the same. After 6 days of heat stress, leaves, roots, and bulk soil were harvested immediately. To collect roots, gently removed *R. simsii* seedlings from the soil, shake off most of the soil, then root was cleaned up with tap water. After the roots were collected, the root surface was sterilized to avoid the influence on the endophytic microbial results. During sterilization treatment, the root system was soaked in 75% alcohol for 1 min, rinsed with sterile water for 3 times, and then used in 2.5% sodium hypochlorite solution for 8 min, and rinsed with sterile water for 3 times again. The last rinse solution was collected and coated on a plate. The surface sterilization was completed when the root system grew sterile. Soil samples used for measuring the physical–chemical properties of soil were mixed by quartering method, screened by 2 mm, and frozen in the refrigerator at −20°C for testing.

### Index determination and method

Plant’s physiological indicators were immediately measured after the leaves were collected. Malondialdehyde (MDA) was determined by thiobarbituric acid method, hydrogen peroxide (H_2_O_2_) was determined by titanium sulfate colorimetric method, Pro was determined by acid ninhydrin colorimetric method, soluble sugar was determined by anthrone colorimetric method, chlorophyll (Chl) content was determined by ethanol extraction colorimetric method, POD activity was measured by spectrophotometer at 470 nm, CAT activity was measured by UV-absorbance method at 240 nm, and APX activity was calculated by measuring the oxidation rate of L-ascorbic acid (Beijing Solarbio Science & Technology Co., Ltd.). UC-visible spectrophotometer (UV-5500, Shanghai Yuan Analysis, China) was used for measurement. Indophenol blue colorimetric method was applied to determine soil ammonium N (NH_4_^+^-N), and ultraviolet spectrophotometry was used for the determination of soil nitrate N (NO_3_^−^-N). This part is the same as our previous research (Liu, L. et al., 2022).

### Sequencing of root endophytic microbial community

The root samples used for sequencing endophytic microbes were submitted to Personal Biotechnology Co., Ltd. (Shanghai, China) for the ITS1 region of ITS rDNA of root endophytic fungi and V5-V7 region of 16S rDNA of root endophytic bacteria for amplification sequencing and library construction. Total genomic DNA from root endophytic microorganisms was extracted using a PowerSoil DNA isolation kit (MO BIO Laboratories, Carlsbad, CA, USA). The quantity and quality of extracted DNA were determined *via* a NanoDrop NC2000 spectrophotometer (Thermo Fisher Scientific, Waltham, MA, USA) and agarose gel electrophoresis, respectively. All samples were pooled in equimolar concentrations and then sequenced on the Illumina MiSeq platform with a paired-end protocol. The primers for fungal sequencing were ITS 1F (5′-CTTGGTCATTTAGAGGAAGTAA-3′) and ITS 2 (5′-GCTGCGTTCTTCATCGATGC-3′). The primers for bacterial sequencing were 779 F (5′-AACMGGATTAGATACCCKG-3′) and 1193 F (5′- ACGTCATCCCCACCTTCC-3′). We used DADA2 Method of QIIME2 software (2019.4, https://docs.qiime2.org/2019.4/tutorials) to filter and quality assessment the sequences. After the consolidation of original data, high-quality reads were divided into operational taxonomic units (OTUs) with 97% similarity.

### Data analysis

Microsoft Excel 2020 was used to sort out the data of plant physiological indexes and soil indexes and then to calculate the mean ± standard deviation (SD) of each index. Four replicates were performed for each treatment. SPSS 17.0 was used for analysis of variance (ANOVA) and Duncan test. Using *R*, alpha diversity and beta diversity of microbial communities were calculated and plotted. We use the Venn Diagram package in *R* to draw Venn diagrams. The heatmap package in *R* is adopted to create a clustered heatmap of the abundance of the top 20 genera according to the data of the mean abundance within the group. The results of the LEfSe analysis consist of two parts, a spectrogram and a histogram of the distribution of LDA values. The Vegen package in *R* was used to map the redundancy analysis (RDA) and to conduct correlation analysis of plant root endophytic microbial communities with plant physiological and soil physical–chemical properties. The significance level was set to *p* < 0.05 for all analyses.

## Results

### Plant physiology and soil physical–chemical properties

**Figure 1:**
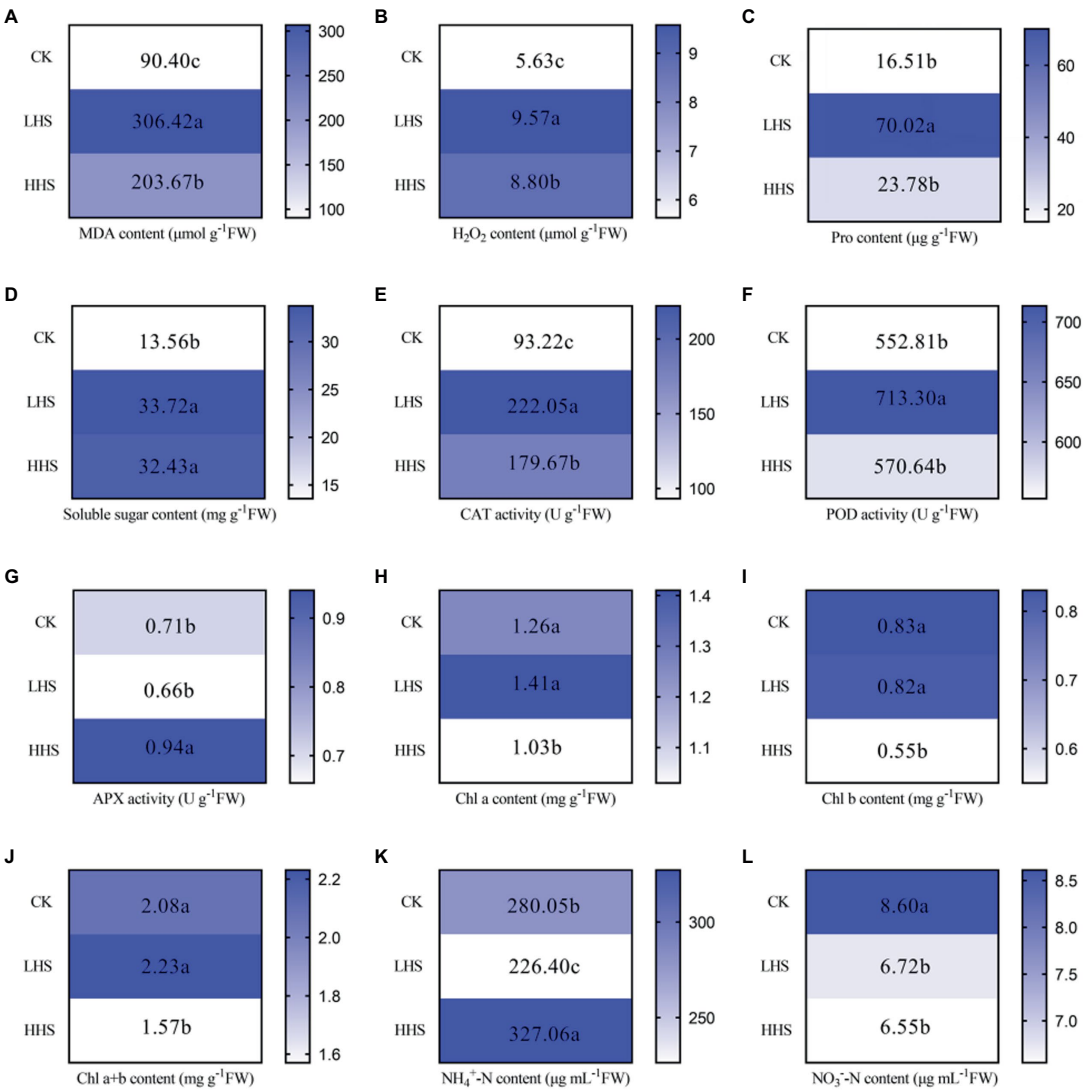
Changes of Rhododendron simsii physiological and soil physical–chemical properties under different temperatures (*n* = 4). **(A)** Change in malondialdehyde (MDA) content; **(B)** Change in hydrogen peroxide (H_2_O_2_) content; **(C)** Change in proline content (Pro); **(D)** Change in soluble sugar content; **(E)** Change in catalase (CAT) activity; **(F)** Change in peroxidase (POD) activity; **(G)** Change in ascorbate peroxidase (APX) activity; **(H)** Change of chlorophyll a (Chl a) content; **(I)** Change of chlorophyll b (Chl b) content; **(J)** Change of chlorophyll a + b (Chl a + b) content; **(K)** Change of soil ammonium nitrogen (NH_4_^+^-N) content; **(L)** Change of nitrate nitrogen (NO_3_^−^-N) content in soil. CK, control; MHS, moderate-heat-stress; HHS, high-heat-stress. Different lowercase represent significant differences between treatments (*p* < 0.05). The data in this section are quoted from our previous study (Liu, L. et al., 2022).

The data in this section are quoted from our previous study (Liu, L. et al., 2022). Compared with CK, MHS and HHS significantly increased the content of MDA (239 and 125%), H2O2 (70 and 56%), soluble sugar (149 and 139%), and the activity of CAT (138 and 93%). Compared with CK, MHS significantly increased Pro content (324%), but HHS had no effect on it. Compared with CK, the activity of POD under MHS was significantly increased by 29%. Compared with CK, HHS significantly increased the activity of APX by 28%. Compared with CK, there was no significant change in chlorophyll content under MHS (*p* > 0.05), but the contents of chlorophyll a (Chl a), chlorophyll b (Chl b), and total chlorophyll (Chl a + b) under HHS were significantly decreased by 22, 51 and 33%, respectively. Compared with CK, MHS significantly decreased soil NH4 + -N by 24%, and HHS increased it by 17%. Compared with CK, MHS and HHS significantly decreased soil NO3--N by 28 and 31%, respectively (Figure 1).

### Root endophytic fungal community

**Figure 2:**
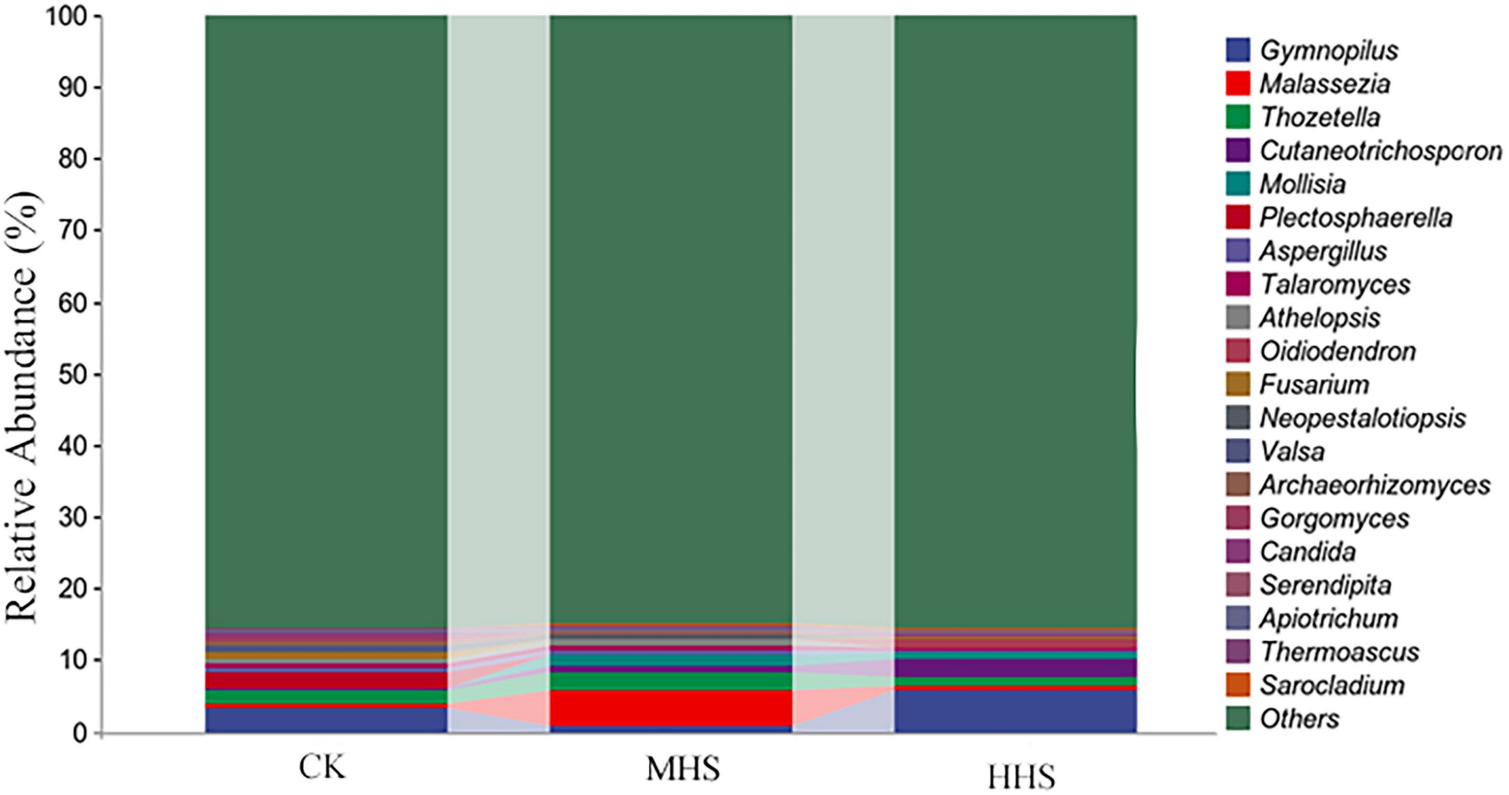
Composition of the top 20 root endophytic fungi communities at genus level. CK, control; MHS, moderate-heat-stress; and HHS, high-heat-stress.

As shown in Supplementary Figure 1A, compared with CK, MHS and HHS did not significantly alter the Chao1, Shannon, and Simpson indices of endophytic fungal alpha diversity in R. simsii roots. There were no significant differences in beta diversity of endophytic fungal community in plant roots between MHS and CK. As for the taxonomic composition of species at genus level, compared with CK, MHS significantly increased Malassezia and Thozetella, and HHS significantly increased Gymnopilus, Cutaneotrichosporon, and Oidiodendron (Figure 2).

**Figure 3:**
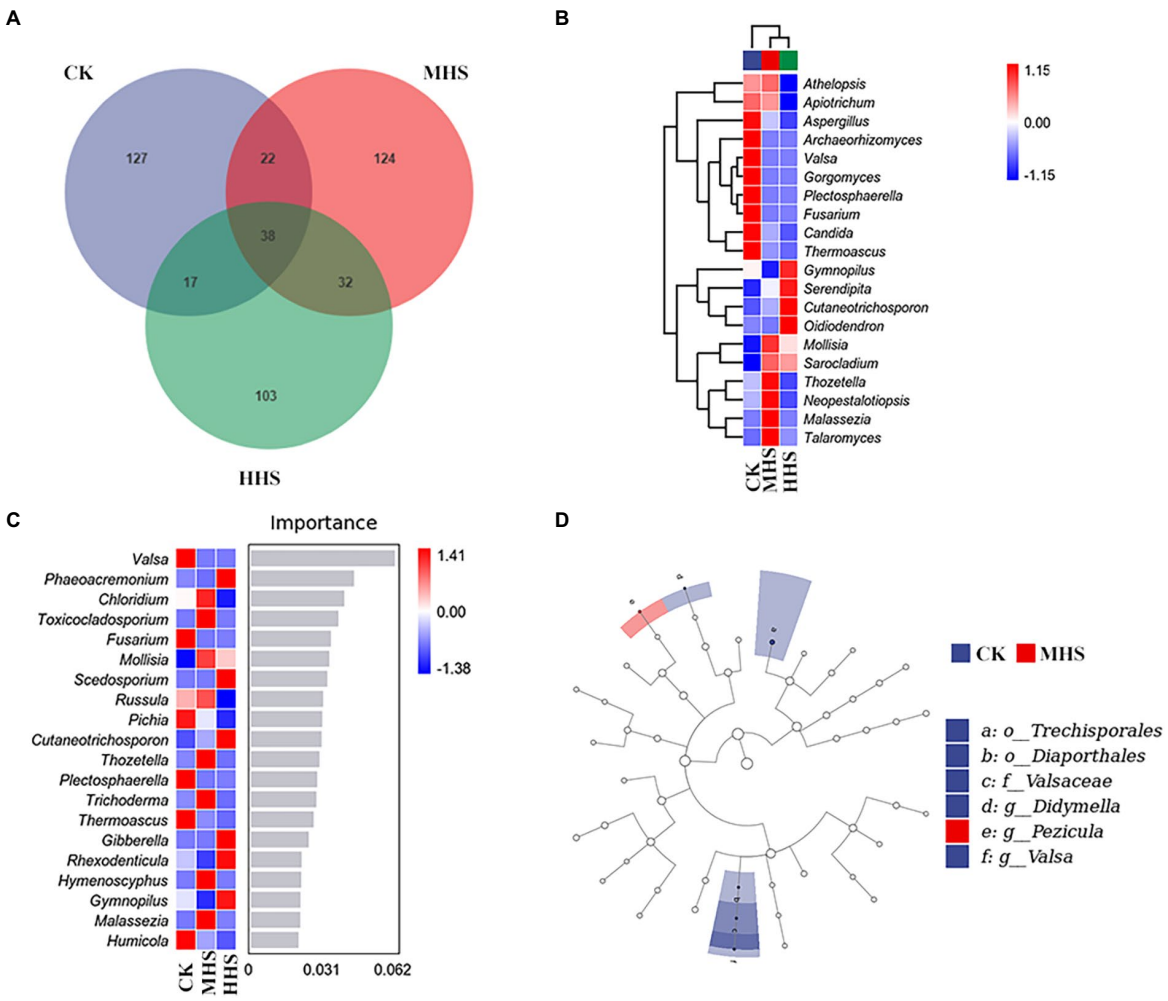
Community characteristics and different genus of endophytic fungi in *R. simsii* roots under different treatments (*n* = 4). **(A)** Venn diagram. **(B)** Heat map of genus composition. **(C)** Random forest model at genus level. **(D)** Phylogenetic distribution of microbial lineages (LDA threshold is 2). CK, control; MHS, moderate-heat-stress; and HHS, high-heat-stress.

As shown in Figure 3A, 204 OTUs were obtained in CK group, 216 OTUs in MHS group, and 190 OTUs in HHS group. Compared to CK, the number of OTUs increased by 5.9% under MHS and decreased by 6.9% under HHS. Also, the proportion of unique OTUs in each group decreased with increasing stress levels. The proportions of unique OTUs under CK, MHS, and HHS were 62.3, 57.4, and 54.2%, respectively (Figure 3A). A heat map of species clustering at genus level can further reveal the effect of heat stress on the community structure of endophytic microorganisms in the root system. The relative abundances of Aspergillus, Archaeorhizomyces, Valsa, Gorgomyces, Plectosphaerella, Fusarium, Candida, and Thermoascus were significantly higher in CK. Mollisia, Sarocladium, Thozetella, Neopestalotiopsis, Malassezia, and Talaromyces showed an evident increase in relative abundance in MHS. The relative abundance of Gymnopilus, Serendipitia, Cutaneotrichosporon, and Oidiodendron increased significantly in HHS (Figure 3B). The analysis of the random forest model at genus level showed that the relative abundances of Valsa, Fusarium and Pichia significantly increased in CK, and relative abundances of Chloridium, Toxicocladosporium, Malassezia, and Rhexodenticula significantly increased in MHS and became dominant strains of this group, and relative abundances of Phaeoacremonium, Scedosporium, and Hymenoscyphus significantly increased and became the dominant strains of the HHS (Figure 3C). LEfSe analysis was used to reveal biomarker that changed significantly between different treatments. More fungal taxa were detected in CK group (2 genera) than in other treatments. At the genus level, Valsa and Didymella were enriched in CK, and Pezicula was enriched in MHS (Figures 3D).

**Figure 4:**
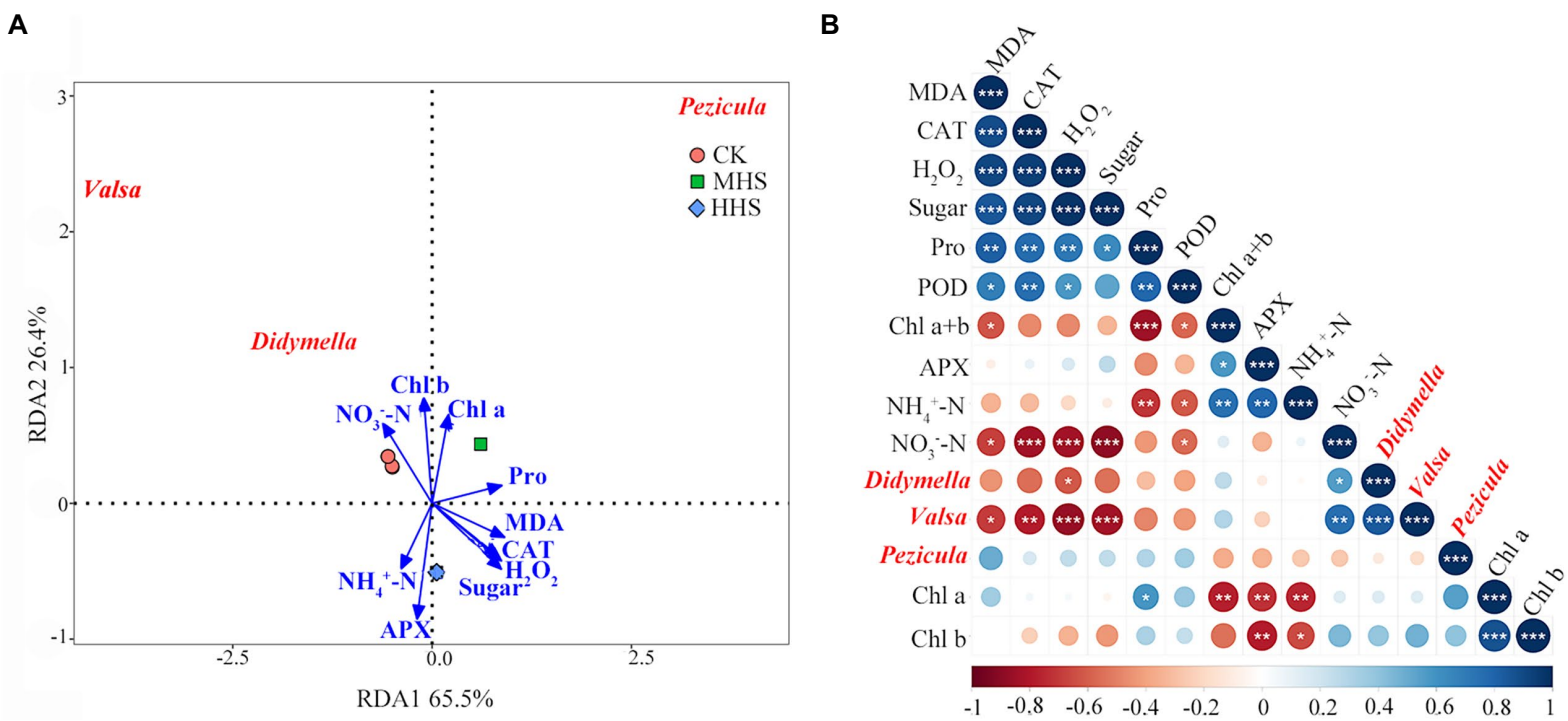
**(A)** Redundancy analysis of significantly up-regulated root endophytic fungal community at genus level and plant physiology and soil available nitrogen (*n* = 4). **(B)** Correlation analysis between plant physiology, soil available nitrogen, and root endophytic fungal community (*n* = 4). Levels of statistical significance are shown with asterisks (*, *p* < 0.05; **, *p* < 0.01; ***, *p* < 0.001). CK, control; MHS, moderate-heat-stress; and HHS, high-heat-stress. MDA, malondialdehyde content; H_2_O_2_, hydrogen peroxide content; Pro, proline content; CAT, catalase activity; POD, peroxidase activity; APX, ascorbate peroxidase activity; Chl a, chlorophyll a content; Chl b, chlorophyll b content; Chl a + b, total chlorophyll content; NH_4_^+^-N, soil ammonium nitrogen content; NO_3_^−^-N, soil nitrate nitrogen content.

Based on the LEfSe analysis, this study correlated the significant enrichment of root endophytic fungal genus with plant physiological indicators and soil physical–chemical properties. The abundance of Didymella was positively correlated with soil NO_3_^−^-N, but negatively correlated with H_2_O_2_ content (*p* < 0.05). The abundance of Valsa was significantly positively correlated with soil NO_3_^−^-N content, but negatively correlated with MDA, H_2_O_2_, and soluble sugar content, and CAT activity in R. simsii leaf (*p* < 0.05). According to RDA, the two axes can be explained together 91.9% of the variation degree of root endophytic fungal community. The abundance of Valsa and Didymella was positively correlated with the content of Chl b and soil NO_3_^−^-N, but negatively correlated with the content of MDA, H_2_O_2_, and soluble sugar, and the activity of CAT. The abundance of Pezicula was positively correlated with Chl a and Pro content, and negatively correlated with NH_4_^+^-N content and APX activity (Figure 4B).

### Root endophytic bacterial community

**Figure 5:**
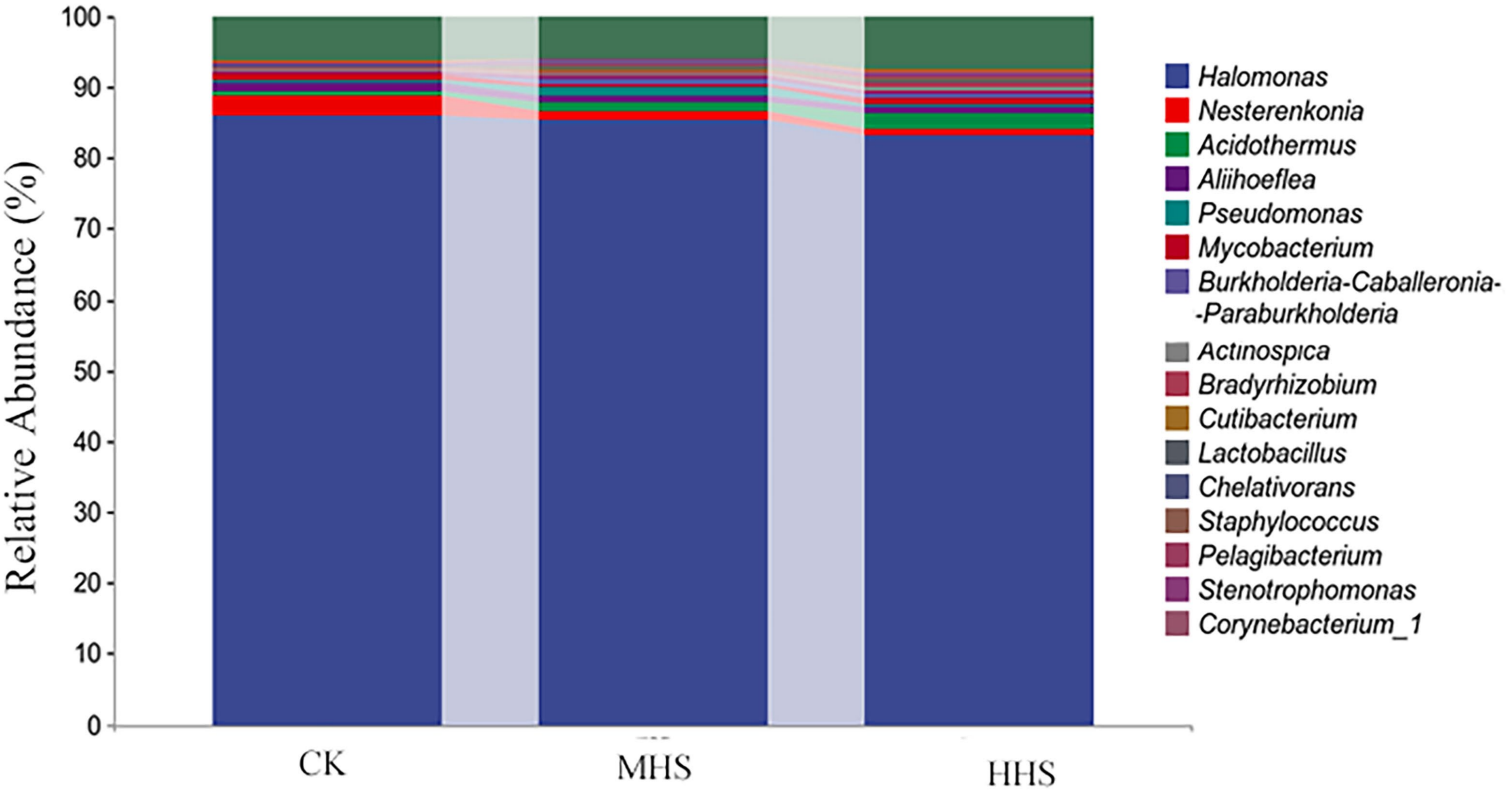
Composition of the top 20 root endophytic bacteria communities at genus level. CK, control; MHS, moderate-heat-stress; and HHS, high-heat-stress.

As shown in Supplementary Figure 2A, compared with CK, MHS and HHS did not significantly alter the Chao1, Shannon, and Simpson indices of endophytic bacterial alpha diversity in R. simsii roots. As seen from the results of the PCoA analysis of beta diversity (Supplementary Figure 2B), there were no significant differences between each treatments. The taxonomic composition of species at the genus level is shown in Figure 5, Malassezia and Thozetella were significantly increased in MHS, Gymnopilus, Cutaneotrichosporon, and Oidiodendron were significantly increased in HHS.

**Figure 6:**
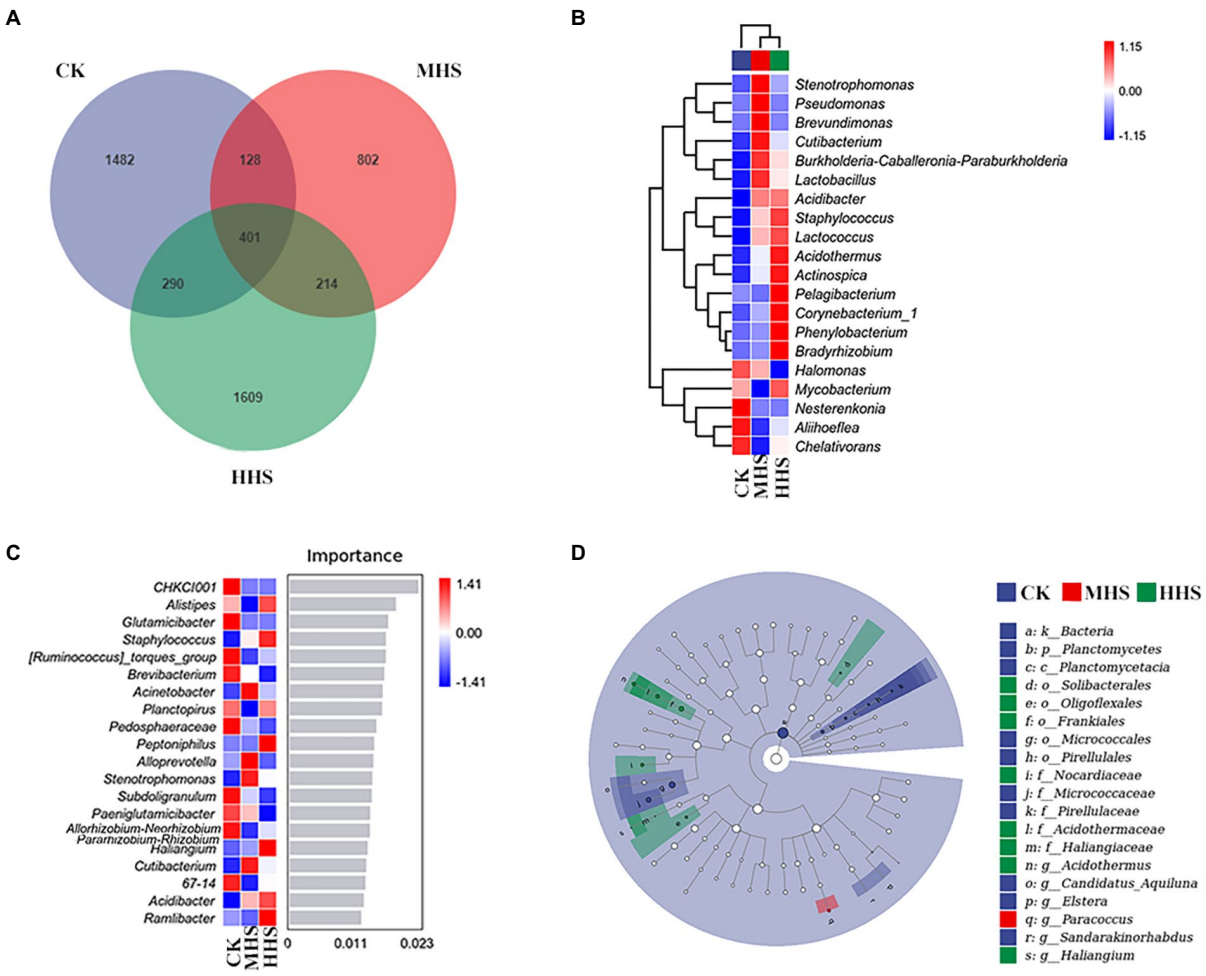
Community characteristics and different genus of endophytic bacteria in *R. simsii* roots under different temperatures (*n* = 4). **(A)** Venn diagram. **(B)** Heat map of genus composition. **(C)** Random forest model of genus level. **(D)** Phylogenetic distribution of microbial lineages (LDA threshold is 2). CK, control; MHS, moderate-heat-stress; and HHS, high-heat-stress.

As shown in Figure 6A, 2,301 OTUs were obtained in CK, 1,545 OTUs in MHS, and 2,514 OTUs in HHS. Compared with CK, the number of OTUs decreased by 32.9% in MHS and increased by 9.3% in HHS. In addition, the proportion of unique OTUs was highest under CK treatment and lowest under MHS. The proportions of unique OTUs under CK, MHS, and HHS were 64.4, 51.9, and 64.0%, respectively (Figure 6A). A heat map of species clustering at genus level can further reveal the effect of heat stress on the community structure of endophytic microorganisms in root. The relative abundance of Halomonas, Nesterenkonia, Aliihoeflea, and Chelativorans significantly higher in CK. Stenotrophomonas, Pseudomonas, Brevundimonas, Cutibacterium, and Burkholderia-Caballeronia-Paraburkholderia showed an increase in relative abundance in MHS. The relative abundance of Acidothermus, Actinospica, Pelagibacterium, Corynebacterium_1, Phenylobacterium, and Bradyrhizobium increased significantly in HHS (Figure 6B). The analysis of the random forest model at genus level shows that the relative abundances of Glutamicibacter, Pedosphaeraceae, Paeniglutamicibacter, and Allorhizobium-Neorhizobium-Pararhizobium-Rhizobium significantly increased in CK, the relative abundances of Acinetobacter, Alloprevotella, Stenotrophomonas, and Cutibacterium significantly increased in MHS and became the dominant strains of this group, and the relative abundances of Staphylococcus, Peptoniphilus, Haliangium, and Acidibacter significantly increased and became the dominant strains of the HHS (Figure 6C). More bacterial taxa were detected in CK (3 genera) than in other treatments. The second most numerous bacterial taxa (2 genera) were detected in HHS. At the genus level, Elstera, Sandarakinorhabdus, and Candidatus-Aquiluna were enriched in CK. Paracoccus was enriched in MHS. Acidothermaceae and Haliangium were enriched in HHS (Figure 6D).

**Figure 7:**
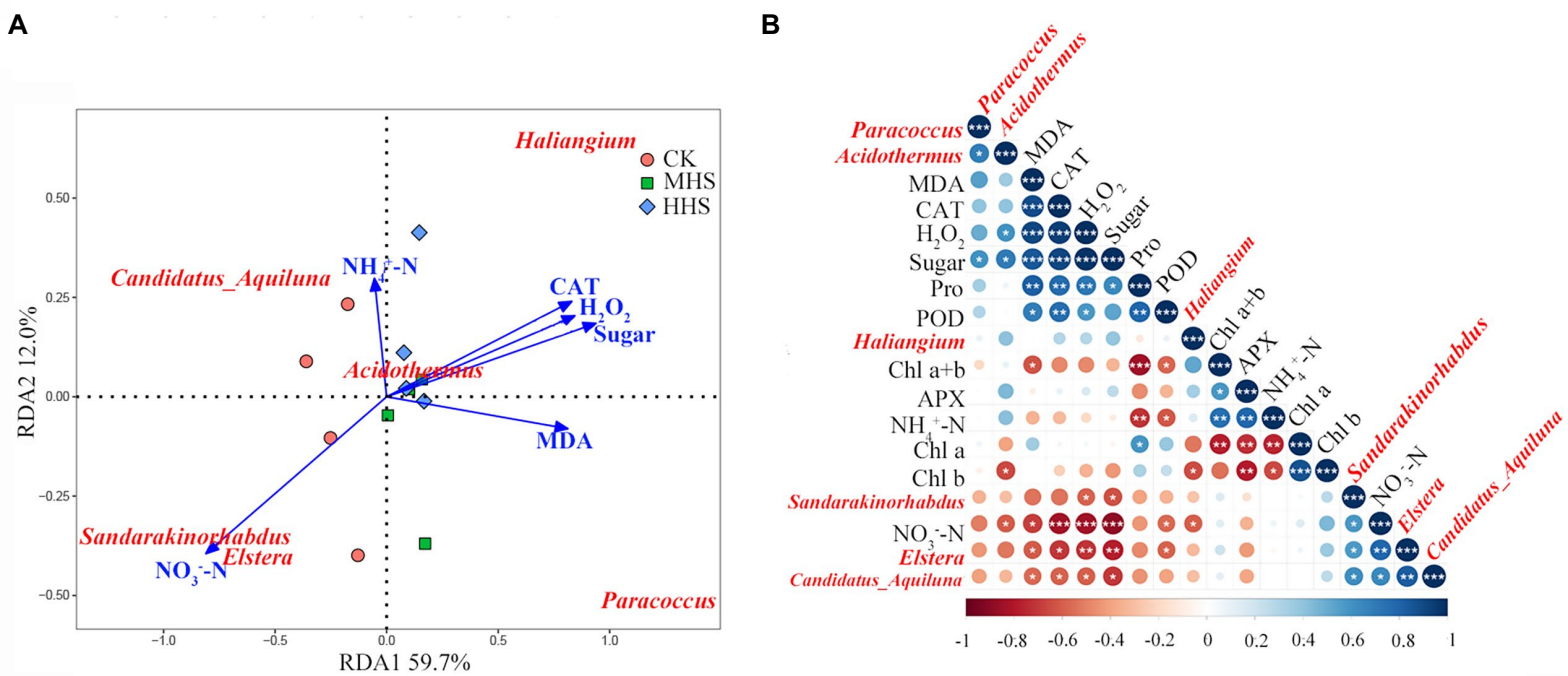
**(A)** Redundancy analysis of significantly up-regulated root endophytic bacterial community and plant physiology and soil available nitrogen content at genus level (*n* = 4). **(B)** Correlation analysis between plant physiology, soil available nitrogen and root endophytic bacterial community (*n* = 4). Levels of statistical significance are shown with asterisks (*p < 0.05, **p < 0.01 and ***p < 0.001). CK, control; MHS, moderate-heat-stress; HHS, high-heat-stress. MDA, malondialdehyde content; H_2_O_2_, hydrogen peroxide content; Pro, proline content; CAT, catalase activity; POD, peroxidase activity; APX, ascorbate peroxidase activity; Chl a, chlorophyll a content; Chl b, chlorophyll b content; Chl a + b, total chlorophyll content; NH_4_^+^-N, soil ammonium nitrogen content; NO_3_^−^-N, soil nitrate nitrogen content.

This study correlated the significant enrichment of microorganisms at the level of the root endophytic bacterial genus with plant physiological indicators and soil physicochemical properties based on the results of the LEfSe analysis (Figure 7). The abundance of Candidatus-Aquiluna positively correlated with soil NO_3_^−^-N content, and negatively correlated with MDA, H_2_O_2_ and soluble sugars content, and CAT activity in R. simsii leaf (*p* < 0.05). The abundance of Sandarakinorhabdus negatively correlated with H_2_O_2_ and soluble sugar content (*p* < 0.05). The abundance of Haliangium positively correlated with soluble protein content (*p* < 0.05). The abundance of Acidothermus correlated with the abundance of Paracoccus (*p* < 0.05; Figure 7A). RDA results show that the common explanatory degree of the first two axes is 71.7%. Sandarakinorhabdus and Elstera positively correlated with NO_3_^−^-N content and were negatively correlated with soluble sugar content, H_2_O_2_ content and CAT activity. MDA content positively correlated with Paracoccus and negatively correlated with Candidatus-Aquiluna. Acidothermus was positively correlated with soil NH_4_^+^-N content (Figure 7B).

### Regulation mechanism of root endophytic microorganism on *Rhododendron simsii* under heat stress

**Figure 8:**
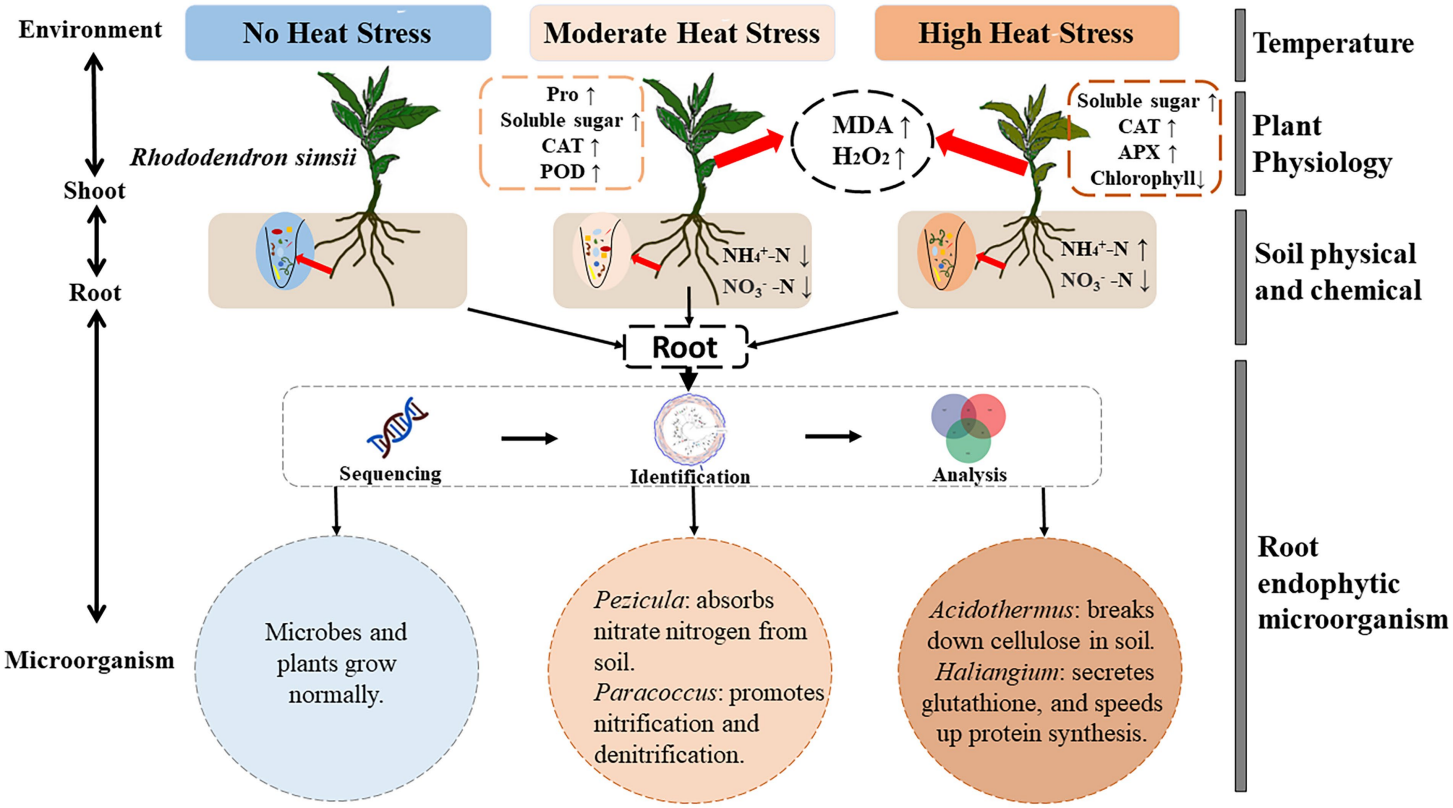
Regulation mechanism of root endophytic microorganism on *R. simsii* under heat stress (*n* = 4). MDA, malondialdehyde content; H_2_O_2_, hydrogen peroxide content; Pro, proline content; CAT, catalase activity; POD, peroxidase activity; APX, ascorbate peroxidase activity; NH_4_^+^-N, ammonium nitrogen content; NO_3_^−^-N, nitrate nitrogen content.

A conceptual figure was developed to summarize the response mechanism of microorganism of R. simsii to heat stress (Figure 8). Under heat stress, beneficial microbial communities in root endophytic microbial communities are enriched. Pezicula helps plants absorb NO_3_^−^-N from soil, Paracoccus promotes N synthesis in soil, and Acidothermus improves soil nutrient content by decomposing cellulose, and Haliangium promotes the synthesis of proteins and other substances in plants. Together, these microbes all play important roles in assisting *R. simsii* to resist heat stress.

## Discussion

### Effects of heat stress on *Rhododendron simsii* physiological and soil physical–chemical indexes

Our study showed that, with the increase level of heat stress, Pro content firstly increased and then decreased, while soluble sugar content increased first and then remained unchanged (Figure 1C). These results suggest that Pro and soluble sugar could regulate the osmotic pressure of plant cells. In addition, CAT and POD showed the strongest activity under MHS, and APX showed the strongest activity under HHS, which could clean up the excessive reactive oxygen species caused by heat stress, which is partly consistent with a previous research (Zhang et al., 2020).

Therefore, our results showed that physiological characteristics of *R. simsii* varied under different heat stress treatment. Heat stress had a significant effect on soil available N content (Figure 1L). Compared with CK, MHS decreased soil available N content, while HHS increased soil NH_4_^+^-N and decreased soil NO_3_^−^-N content (Figure 1L). Moreover, the rapid action of soil microorganisms may bring changes to available N contents, which accelerated the oxidation of soil organic N (Ashraf et al., 2019; Wen et al., 2019; Chen et al., 2020). Similarly, our previous study found that heat stress significantly increased the abundance of soil bacteria such as Burkholderia-Caballeronia-Paraburkholderia, Occallatibacte and soil fungi such as Candida, Mortierella and Boothiomyces, which may have changed NH_4_^+^-N content (Liu, N. et al., 2022).

### Effects of heat stress on root endophytic fungal communities

Although there was no significant difference in alpha diversity between different treatments, some differences were observed in endophytic fungal community composition (Figure 2). Russula is a group of ectomycorrhizal fungi, which is abundant in polysaccharides, fatty acids, proteins, sesquiterpenes, and other active substances (Caboň et al., 2017). Previous studies have found that Russula may promote growth and inhibit pathogenic bacteria (Luo et al., 2019). The abundance of Russula increased under MHS suggested that the genus may be able to help plants resist heat stress. Phaeoacremonium is a common pathogen that can cause grape blight (Aroca et al., 2010). Cutaneotrichosporon was considered to be a widely distributed saprophytic fungus, and mainly existed in soil (Bracharz et al., 2017). Phaeoacremonium and Cutaneotrichosporon increased in the presence of HHS, probably due to heat environment. In general, heat stress changes the community structure of root endophytic fungi.

According to our LEfSe analysis (Figures 3D), correlation analysis (Figure 4A), and RDA (Figure 4B) results, the abundance of Valsa and Didymella was positively correlated with soil NO_3_^−^-N, and negatively correlated with MDA, H_2_O_2_, Pro, and soluble sugar contents in leaves, respectively. Studies have shown that both Valsa and Didymella are plant pathogens, which may cause plant wilt and death. Heat treatment significantly reduced the abundance of Valsa and Didymella, and it is possible that the beneficial microorganisms in plant roots increased significantly at high temperature, which inhibited the plant’s growth. The abundance of Pezicula negatively correlated with soil available N content and positively correlated with the content of MDA, Chl a, and Chl b (Figure 7B). It may because *R. simsii* likely to use NO_3_^−^-N (Wei et al., 2016). Under MHS, the abundance of Pezicula was significantly increased and it was able to utilize a large amount of soil NO_3_^−^-N. Therefore, the NO_3_^−^-N content was reduced. There are few reports about Pezicula and its metabolites. Studies have shown that Pezicula strains have the potential to synthesize a variety of bioactive secondary metabolites, which has important research and application prospects for sterilization and weed control (Terhonen et al., 2016). In addition, *Pezicula ericae* is a mycorrhizal fungus that has been discovered and confirmed to have a broad spectrum of mycorrhizal fungi, previous studies have shown that Pezicula ericae can significantly improve the ability of Rhododendron spp. to alleviate abiotic stress (Lin et al., 2019; Song et al., 2020). Combined with the results of this study, we also believe that the significant enrichment of Pezicula in MHS may play a vital role in plant resistance to heat stress.

### Effects of heat stress on root endophytic bacterial communities

In this study, some differences in endophytic bacterial community composition have been observed (Figure 5). The abundance of both Acinetobacter and Stenotrophomonas increased significantly under MHS. Acinetobacter is a rhizosphere growth-promoting bacterium with phenolic acid degradation function, which can alleviate crop continuous cropping disorder and is also a very common bacterium in flowers (Arif et al., 2020). Stenotrophomonas is a group of Gram-negative aerobic fungi that secrete antifungal compounds, it synthesizes disease-resistant extracellular enzymes, and produce osmoregulatory substances that promote growth, and have resistance to heavy metals (An and Berg, 2018). These results suggest that Acinetobacter and Stenotrophomonas may be beneficial microorganisms in R. simsii roots that help plants to resist heat stress.

According to LEfSe analysis (Figure 6), correlation analysis (Figure 7A), and RDA results (Figure 7B), Paracoccus significantly enriched under MHS, while Acidothermus and Haliangium enriched in HHS. Paracoccus and Acidothermus were positively correlated with leaf soluble sugar content. Meanwhile, Acidothermus was significantly negatively correlated with Chl b content in leaves and NO_3_^−^-N content (Andrews et al., 2013). Since Acidothermus prefers a thermal environment, NO_3_^−^-N is absorbed and utilized in large quantities to transform into protein and other nutritional elements. However, as HHS has destroyed plant light and systems, Chl b levels have declined significantly. Studies have proved that Paracoccus is an environmental-friendly α proteobacteria strain, has good N removal ability, promotes nitrification and back nitrification, and can improve the tolerance of plants to heavy metals and salt stress (Olaya-Abril et al., 2021). Acidothermus is a genus of thermophilic acidophilic bacteria. Studies have found that thermophilic Acidothermus can breaks down cellulose in soil and improve soil fertility (Wang et al., 2015). In this study, the abundance of Acidothermus in soil was significantly decreased, while the abundance of Acidothermus in roots was significantly increased under heat stress, which may be due to the fact that heat stress accelerates the infection of the genus bacteria in soil into the root system, which better helps plants to utilize and decompose organic matter, so as to improve the heat tolerance of plants to heat stress. The abundance of Elstera and Sandarakinorhhabdus significantly positively correlated with soil NO_3_^−^-N, and negatively correlated with CAT, H_2_O_2_, and soluble sugar content in leaves. The reason for this may be that with the increase in temperature, other endophytic bacteria in root system proliferated, to utilize NO_3_^−^-N, and transfer nutrients to plant, leading to decrease in soil NO_3_^−^-N content. It has been proved that Haliangium can increase the resistance of plants to salt stress (Zhao et al., 2021). The abundance of Haliangium was positively correlated with leaf soluble protein content. Some research suggests that under heavy metal stress, the abundance of Haliangium increases significantly, which may help plants mitigate the effects of stress (Morya et al., 2020; Bian et al., 2021). Accordingly, Shrivastava and Sharma (2021) suggested that it ameliorates oxidative stress response in plants by increasing intracellular glutathione levels. This is consistent with our findings that in HHS, where the increased abundance of this genus tries to help plants resist heat stress.

### Regulation mechanism of root endophytic on *Rhododendron simsii* under heat stress

In summary (Figure 8), our results showed that heat stress induce significantly alterations in endophytic microbial community of root system, with an increase in Pezicula and Paracoccus under MHS, and an increase in Acidothermus and Haliangium under HHS, respectively. Pezicula ericae in the genus of Pezicula is considered one of the Ericoid mycorrhizal fungi that can obtain N and phosphorus from complex organic matter to meet its own growth needs, as well as to transfer nutrients to the plant to promote its growth and development and to resist stress (Walker et al., 2011). Another species of this genus, Pezicula neosporulosa, is involved in the metabolism of carbohydrates and the synthesis of enzymes that affect the secondary metabolites of plant and can provide nutrients to plant (Yuan et al., 2022). In addition, fungi within genus Acidothermus have been shown to hydrolyze insoluble digestible cellulose and produce soluble products, that can use trifluoroacetic acid to hydrolyze their products into monosaccharides (Liu, N. et al., 2022). Moreover, Paracoccus, a heterotrophic nitrification-aerobic denitrification bacterium, which has been studied in recent years that plays an important role in N cycling (Chen et al., 2020). Paracoccus can transform NH_4_^+^-N into NO_3_^−^-N, which can be directly absorbed and utilized by plants, and converted into proteins, chlorophyll, amino acids and other substances needed by plants (Jaffer et al., 2019). The bacteria of this genus were significantly enriched under MHS, which may promote the utilization of soil NH_4_^+^-N. The soil NH_4_^+^-N content was significantly reduced and was absorbed and utilized by plants to resist high temperature stress. Haliangium is also known to degrade complex organic compounds (Maarastawi et al., 2018). This genus of bacteria is widely regarded as beneficial and plays a significant role in plant resistance to abiotic stress (Wang et al., 2020). Therefore, under heat stress, endogenous fungal and bacterial communities of the plant root system have been changed, which indicate that the beneficial flora is significantly enriched. Soil organic matter is decomposed as inorganic nutrients can be used by plants that help plants to absorb NO_3_^−^-N. These inorganic nutrients are used in the root system for the purpose to combine with infiltration regulatory substances, such as protective enzymes, etc. to help plants to resist heat stress.

## Conclusion

Heat stress can cause changes in plant physiological indexes, soil available N content, and root endophytic microbial community structure. In response to MHS, plants remove excessive reactive oxygen species by increasing the concentration of osmoregulatory substances and the activity of protective enzymes. Under MHS, the endophytic fungi *Pezicula* and the endophytic bacteria *Paracoccus* were significantly enriched, while under HHS, the endophytic bacteria A*cidothermus* and *Haliangium* were significantly enriched, which were negatively correlated with NO_3_^−^-N content. Therefore, our results suggest that heat stress may affect the root endophytic microbial composition, which will help promote the absorption of NO_3_^−^-N in *R. simsii*, and affect the physiological indicators of the plant. Our findings highlight the importance of understanding the mechanism underlying the interactions between various endophytic microorganisms and plants.

## Data availability statement

The datasets presented in this study can be found in online repositories. The names of the repository/repositories and accession number(s) can be found at: https://www.ncbi.nlm.nih.gov/, PRJNA859367 and PRJNA859369.

## Author contributions

LG and WL designed this study. LL, JL, and XT conducted the experiments and wrote the original manuscript. PW and XianC organized the original data. JS, YZha, and LZ guided to the method of data analysis. BW, SL, SW, XiaoC, and YZho modified the original manuscript. LG acquisition fund. All authors contributed to the article and approved the submitted version.

## Funding

This work was supported by the Hainan Provincial Natural Science Foundation of China, grant number (320RC470 and 2019RC111), and Priming Scientific Research Foundation of Hainan University [KYQD(ZR)1982].

## Conflict of interest

The authors declare that the research was conducted in the absence of any commercial or financial relationships that could be construed as a potential conflict of interest.

## Publisher’s note

All claims expressed in this article are solely those of the authors and do not necessarily represent those of their affiliated organizations, or those of the publisher, the editors and the reviewers. Any product that may be evaluated in this article, or claim that may be made by its manufacturer, is not guaranteed or endorsed by the publisher.
