# Supplementary figures and images for: Changes of endophytic microbial community in Rhododendron simsii roots under heat stress and its correlation with leaf physiological indicators
Source: Front Microbiol. 2022 Nov 17;13:1006686. doi: 10.3389/fmicb.2022.1006686 (PMC9712210; doi:10.3389/fmicb.2022.1006686)

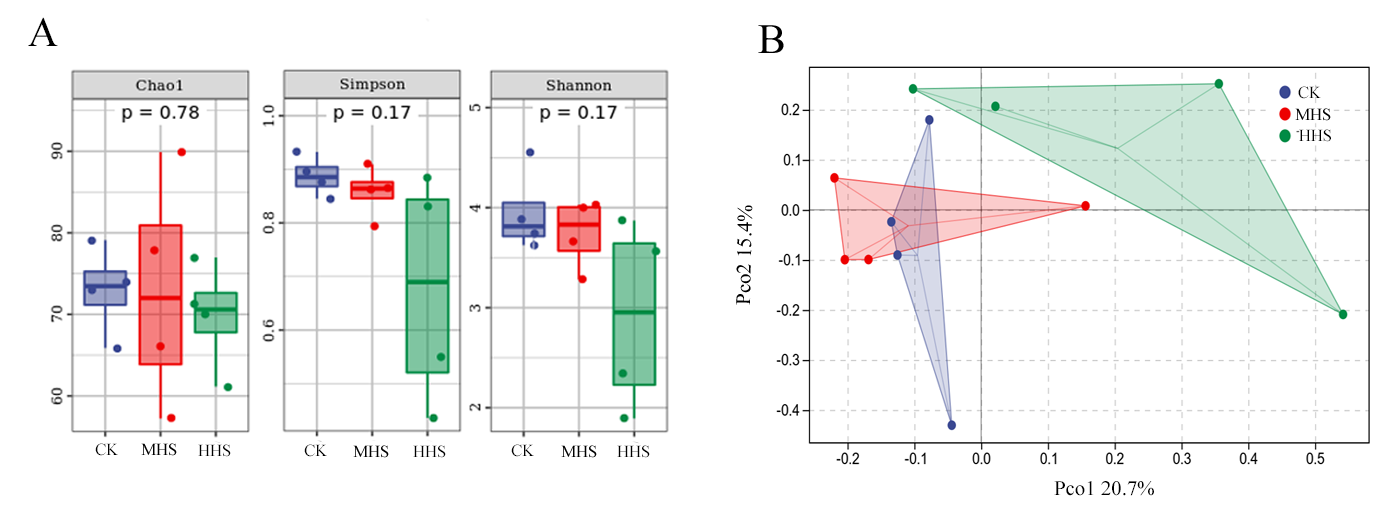

Supplement: Supplementary file 1 [file Image_1.TIF]

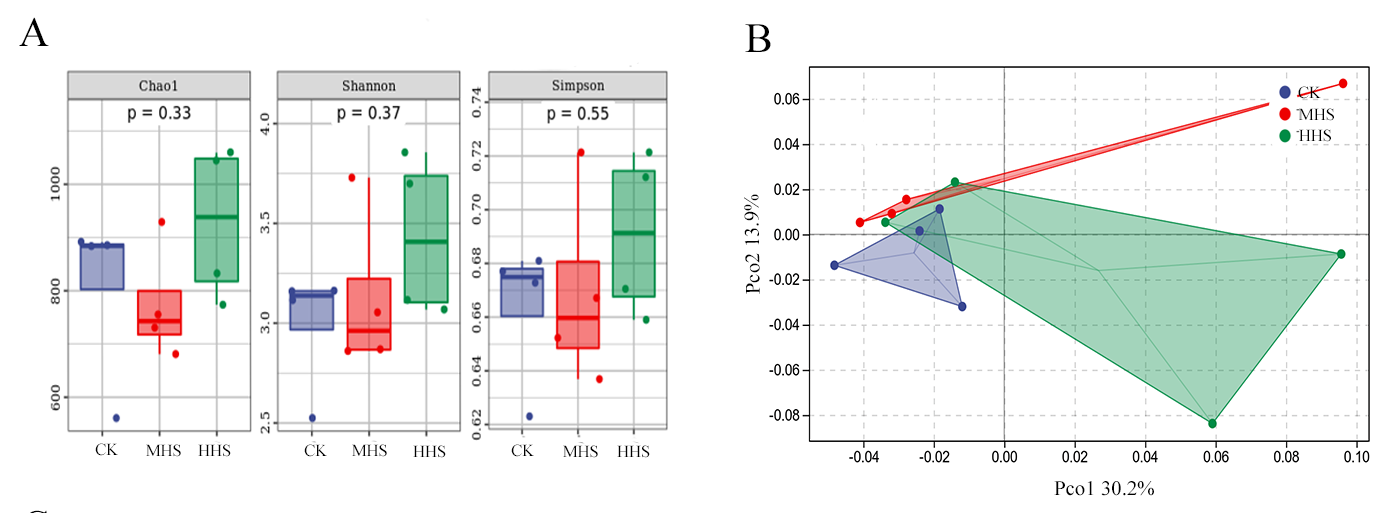

Supplement: Supplementary file 2 [file Image_2.TIF]
